# Organic Nanocarriers for Bevacizumab Delivery: An Overview of Development, Characterization and Applications

**DOI:** 10.3390/molecules26144127

**Published:** 2021-07-07

**Authors:** Aline de Cristo Soares Alves, Franciele Aline Bruinsmann, Silvia Stanisçuaski Guterres, Adriana Raffin Pohlmann

**Affiliations:** Programa de Pós-Graduação em Ciências Farmacêuticas, Universidade Federal do Rio Grande do Sul, Porto Alegre 90610-000, Brazil; fbruinsmann@gmail.com (F.A.B.); silvia.guterres@ufrgs.br (S.S.G.)

**Keywords:** antibody, nanotechnology, controlled release, ocular diseases, anticancer activity

## Abstract

Bevacizumab (BCZ) is a recombinant humanized monoclonal antibody against the vascular endothelial growth factor, which is involved in the angiogenesis process. Pathologic angiogenesis is observed in several diseases including ophthalmic disorders and cancer. The multiple administrations of BCZ can cause adverse effects. In this way, the development of controlled release systems for BCZ delivery can promote the modification of drug pharmacokinetics and, consequently, decrease the dose, toxicity, and cost due to improved efficacy. This review highlights BCZ formulated in organic nanoparticles providing an overview of the physicochemical characterization and in vitro and in vivo biological evaluations. Moreover, the main advantages and limitations of the different approaches are discussed. Despite difficulties in working with antibodies, those nanocarriers provided advantages in BCZ protection against degradation guaranteeing bioactivity maintenance.

## 1. Introduction

Bevacizumab (BCZ) is a recombinant humanized monoclonal antibody against the vascular endothelial growth factor (VEGF) [1,2,3,4]. This full-size human immunoglobulin G1 (C_6538_H_10034_N_1716_O_2033_S_44_, 149 kDa) binds to all isoforms of VEGF and, consequently, blocks the interaction with the VEGF receptors [4,5,6]. VEGF, a multifunctional protein, induces the proliferation of vascular endothelial cells during embryonic development, physiological and pathological angiogenesis [1,3,7]. Angiogenesis is a process in which the formation of new blood vessels occurs from a pre-existing vascular endothelium [2,8]. Pathologic angiogenesis is observed in several diseases such as vascular malformations, atherosclerosis, obesity, arthritis, ophthalmic diseases, and cancer [9,10].

Among the ophthalmic diseases, VEGF is overexpressed in diabetic retinopathy, corneal neovascularization, macular edema, age-related macular degeneration, and neovascular glaucoma [3,11]. In these instances, angiogenesis can cause a block of light, corneal scarring, edema, high intraocular pressure, impaired visual acuity, and inflammation [3,11]. Moreover, angiogenesis is involved in the development of tumors [12]. VEGF is overexpressed in the majority of cancers, been associated with chemoresistance and poor prognosis compared with negative VEGF tumors [13,14,15].

In this perspective, BCZ (Avastin^®^, Genentech, South San Francisco, CA, USA) was the first anti-VEGF drug approved by the U.S. Food and Drug Administration (FDA) in 2004, for intravenous treatment of metastatic colorectal cancer combined with chemotherapy [15,16,17]. Afterward, its use was approved for the treatment of metastatic recurrent non-squamous non-small cell lung cancer [18], advanced renal cell carcinoma [19], and recurrent glioblastoma multiforme as monotherapy [20]. BCZ is not approved by FDA for the treatment of ocular diseases; however, its intravitreal administration has expanded widely on an off-label basis [4,11,21].

The BCZ mechanism of action is based on the neutralization of VEGF. In ocular tissues, the mitogenic activity of endothelial cells is inhibited resulting in a decrease of vascular permeability. In tumoral tissue, BCZ induces a hypoxia state and blocks the mechanism that promotes and sustains the new vessel growth. In this way, the tumor vasculature is normalized, and the aberrant and immature vascular proliferation is suppressed [5,22,23]. These effects result in the apoptosis of tumor endothelial cells and a reduction in the intratumoral interstitial fluid pressure [24]. The levels of circulating VEGF are increased as a mechanism of compensatory upregulation; however, it is not sufficient to induce angiogenesis [23].

The pharmacokinetic profile of BCZ by intravenous infusion is characterized by a two-compartmental model and first-order elimination [24,25]. A single dose of BCZ shows a dose-response relationship [26]. BCZ has linear pharmacokinetics in the range of 0.3–10 mg kg^−1^, a low clearance rate, a limited volume of the central compartment, and a serum BCZ half-life of 13–21 days [12,25,26]. The administration of a dose of 10 mg kg^−1^ every 14 days result in an accumulation ratio of 2.8 with steady-state concentrations in about 100 days [24,26]. The characteristics such as tumor burden, sex, body weight, and albumin levels can modify the clearance [26].

Clearance of intravitreal BCZ with a short half-life of 4.9–9.8 days has been reported [27,28,29], being thus necessary multiple injections to maintain the therapeutic effect [11]. The repeated injections can cause some local effects like intravitreal and retinal hemorrhage, retinal detachment, endophthalmitis, and cataract [30]. The intravenous BCZ is generally well-tolerated, but some serious and unusual complications are noted including wound dehiscence, gastrointestinal perforation, high-grade thrombosis, bleeding, proteinuria, and hypertension [23,31].

In this way, the development of controlled release systems for BCZ is an interesting strategy to overcome the limitations mentioned above. The use of nanoparticles as colloidal carriers can promote alterations in drug pharmacokinetics and, consequently, decrease the dose, toxicity, and cost due to improved efficacy [32,33,34]. Moreover, antibodies formulated in nanoparticles can present slower enzymatic degradation [32].

**Figure 1:**
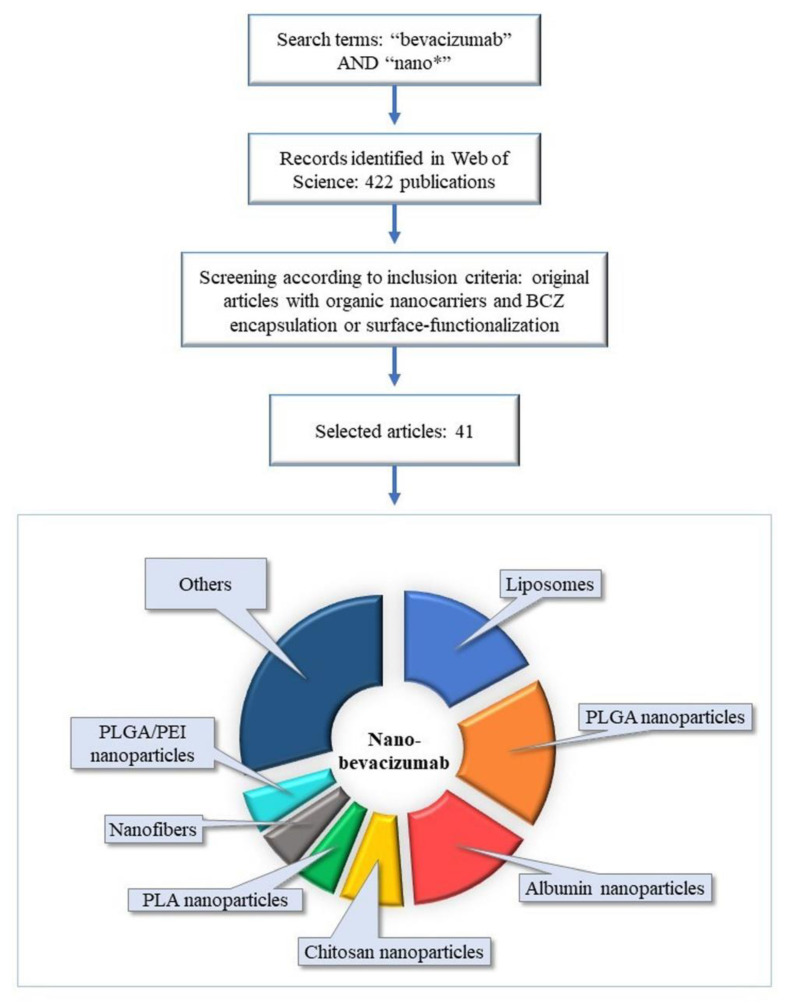
Schematic illustration of search methodology of articles involving the current approaches for BCZ delivery using organic nanocarriers. Abbreviations: BCZ, bevacizumab; PEI, polyethylenimine; PLA, polylactic acid; PLGA, poly(lactic-co-glycolic acid).

Inorganic, organic, and hybrid nanocarriers are named depending on the chemical nature of their materials. Therefore, this review comprises an overview of the current approaches for BCZ delivery using organic nanocarriers. The methodology was based on a search performed on the database Web of Science, on the 3 July 2020 and more recently for an update on the 1 February 2021 (Figure 1). We crossed “bevacizumab” AND “nano*” by topic, resulting in 422 publications. The inclusion criteria were organic nanocarriers and BCZ encapsulation or surface-functionalization resulting in 41 original articles (Table 1). The results from these original articles were analyzed and detailed in subsequent sections. Additionally, other articles were used in the present review to introduce and better discuss the issue.

## 2. Development of Nanoparticulate Drug Delivery Systems for BCZ

Organic nanocarriers are in general biocompatible and biodegradable. Different materials can be used to build those nanocarriers, including lipids, polymers, surfactants, proteins, and polysaccharides. In general, non-covalent interactions maintain the nanostructure dispersed in liquid, semisolid or solid matrices. Nevertheless, in some cases, covalent bonds are used to obtain drug-adjuvant conjugates, which are administered as a colloidal solution. Several types of systems have been developed in the past 50 years, such as lipid-based nanocarriers, polymeric nanoparticles, polysaccharide-based nanoparticles, albumin nanoparticles, polymeric micelles, and polymer-drug conjugates [34,76].

Antibodies can be delivered at a specific site after being attached to or encapsulated, absorbed, entrapped, and dissolved in the nanocarrier [76]. An appropriate physicochemical characterization is necessary to guarantee the quality of formulations. Size distribution, surface characteristics (negative, neutral, or positive), shape (spherical or fibers), drug content, loading capacity, and entrapment efficiency (EE%) as well as drug release, and physicochemical stability are evaluated during pre-formulation development [77,78].

The size and surface characteristics of nanoparticles interfere in their biological behaviors, pharmacokinetic profiles, and tissue distribution [79,80]. In general, the nanoparticles should have a hydrodynamic diameter higher than 100 nm to avoid renal clearance and lower than 300 nm to avoid the cellular uptake by the mononuclear phagocyte system [81,82]. Ideally, the particle size distribution should be unimodal with low polydispersity index (PDI). PDI represents the size dispersity of a sample [83,84].

The surface potential of nanoparticles can influence the cellular internalization and, as a consequence, their permanence time in blood circulation [83,84]. In addition, the physicochemical stability is directly influenced by the surface potential. Absolute values of ζ potential higher than 30 mV predict better stability of colloidal dispersions, when the mechanism of stability is based on the electrostatic repulsion [84,85].

**Figure 2:**
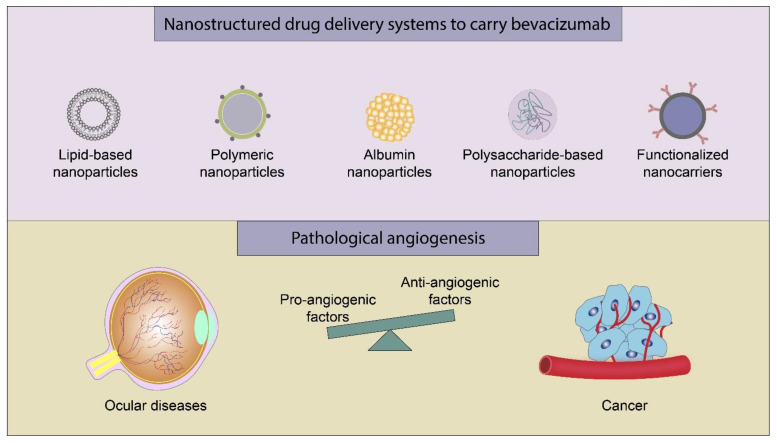
Main strategies for delivery of BCZ and biological applications.

Concerning BCZ nanocarrier formulations, we summarize the physicochemical characteristics (mean size, PDI, ζ potential, and EE%) in Table 2. In the sub-sections below, we describe the different BCZ-nanocarriers reported in the literature: lipid-based nanocarriers, polymeric nanoparticles, polysaccharide-based nanoparticles, albumin nanoparticles, and other nanoparticles (Figure 2). In general, the end dosage form consisted of nanocarrier dispersed in water (liquid); however, freeze-dried particles (dry powder) and nanogel (semisolid) were also produced.

### 2.1. Lipid-Based Nanoparticles

The lipid-based nanocarriers are biocompatible and biodegradable showing very low or no toxicity [86,87]. Liposomes, solid lipid nanoparticles, and nanoemulsion are examples of these nanocarriers, been used to encapsulate BCZ [86,87]. Liposomes (multilamellar vesicles) obtained using egg phosphatidylcholine and cholesterol showed BCZ entrapment efficiency of 45.5% [35], while liposomes of phosphatidylcholine, phosphatidylserine, and cholesterol (unilamellar vesicles, *d* = 163 nm) containing BCZ and annexin A5 showed negative ζ potential (−7.2 ± 0.6 mV) and EE% about 25% [36]. More recently, BCZ-loaded liposomes prepared with phosphatidylcholine and cholesterol (*d* = 152 nm) showed ζ potential of −22.6 mV and entrapment efficiency close to 37% [37]. In this case, BCZ showed an in vitro release profile with an initial burst phase (21.1%) followed by slow diffusion and degradation, totalizing 55.1% of BCZ released after 48 h.

Neutral liposomes obtained using 1,2-dipalimitoyl-Sn-glycero-3-phosphocholine and cholesterol (*d* = 141 nm, ζ potential = −0.4 mV) showed spherical shape with a smooth surface, and a stability concerning size over 2 weeks. BCZ entrapment efficiency was close to 50% showing in vitro release at different proportions when surfactants were added to the medium [12.9%, sodium dodecyl sulfate; 19.4%, tween-20; 4.6%, triton X-100; and 20.6%, poly(ethylene glycol)-2000 (PEG-2000)]. Interestingly, encapsulated BCZ maintained structural stability against thermal stress (4, 24, 37, and 50 °C) [38].

Echogenic liposomes that amplify echo signals after ultrasound energy application are produced by rehydration-lyophilization, which method associates air to the lipid providing the ultrasound reflectivity [88,89]. In this way, echogenic BCZ-loaded liposomes (*d* = 893 nm) were produced as a targeted ultrasound contrast agent with an EE% of 32.1 ± 6.6%, of which 60% of encapsulated BCZ was able to bind to its antigen at the same affinity as the free BCZ. A in vitro release assay revealed that about 30% of BCZ was released from liposomes after 30 min in phosphate-buffered saline (PBS) and 54% after 60 min in porcine plasma. An immediate release of antibody (80 μg of BCZ per 5 mg of lipid) in human plasma has been observed using color Doppler ultrasound [39].

Photodynamic therapy has been used in combination with BCZ as an effective treatment of ocular diseases and cancer [90,91]. BCZ-liposomes (EE% = 60–80%) containing benzoporphyrin derivative monoacid A had mean size of 120 ± 9 nm and positive ζ potential (+15 mV) since 1,2-dioleoyl-3-trimethylammonium-propane has been used to formulate the vesicles. BCZ has been released in 85% in human serum. Furthermore, after 60 days of storage at 4 °C in nitrogen, mean size, polydispersity index, and ζ potential were constant demonstrating the kinetic stability of the formulation [40].

Solid lipid nanoparticles (SLN), stabilized by surfactants in water, are composed of solid lipids at body temperature [87,92]. SLN can be used to carry poorly-water soluble drugs with solubility in the lipid phase [86] or hydrophilic drugs when chemically attached to a component of the lipid phase [92]. A BCZ conjugate was obtained by hydrophobic ion-pairing technique to improve the BCZ incorporation into stearic acid SLN [41]. The final formulation using stearic acid (*d* = 515 nm) showed a spherical shape, rugged surface, and an EE% lower than 30%. In physiologic pH conditions, no BCZ was released from SLN until 48 h [41]. Similarly, BCZ was conjugated by ion-pairing using a commercial lipid (Intralipid^®^) to form a nanoemulsion [42]. Besides BCZ (EE% = 47%), the formulation carried temozolomide and rapamycin in association. The nanoemulsion (*d* = 262 nm) showed negative surface (−33.1 mV) and kinetic stability at different pH conditions (5.0, 5.6, 6.0, and 7.4) [42].

### 2.2. Polymeric Nanoparticles

Polymeric nanoparticles are composed of biocompatible polymers. In some cases, the nanoparticles are formulated using biodegradable polymers such as poly(lactide) (PLA), poly(lactide-co-glycolide) (PLGA), and poly(ε-caprolactone) (PCL) [92,93]. To reduce immunological and intermolecular interactions with the surface, the nanoparticles are coated with nonionic surfactants [87]. BCZ-loaded PLGA nanoparticles (*d* = 133 to 199 nm) have been developed in many studies showing spherical shape, smooth surface, and negative ζ potential [43,45,46] (Table 1 and Table 2).

Comparing the release studies, we can observe different behavior in each case. In PBS, BCZ release was less than 10% after 21 days, while in rabbit vitreous (ex vivo), BCZ showed an initial burst release of about 10% and, after 6 weeks, the release reached 49% [43]. The particles size and ζ potential remained constant during the drug release process after 28 and 21 days, respectively. In another study, BCZ release in PBS showed an initial burst (>40% after 2 h) with a sustained and slow-release (about 40% released within 7 days followed by a slow-release until 21 days) [45]. Nanoencapsulated BCZ showed a pH-dependent release profile when assayed at pH 6, 7.4, and 10 [46].

An important parameter to be evaluated in the BCZ encapsulation process is the maintenance of antibody structure. BCZ loaded in PLGA nanoparticles underwent a structural change after encapsulation and lyophilization process [46]. To overcome this problem, in a further study, trehalose has been added to this formulation [47] with no interference in the BCZ EE% (88 ± 5%). Further, greater preservation of BCZ secondary and tertiary structures was observed in comparison to other formulations without trehalose [47].

BCZ-loaded PLGA nanoparticles were coated with chitosan (*d* = 222 nm; EE% 69.3%), resulting in a positive ζ potential (+32.8 mV). A slow and controlled BCZ release profile has been observed (<25% after 72 h) in PBS. The in vitro permeation using goat sclera as membrane and PBS as receiver medium revealed a flux higher for the nanoencapsulated BCZ, combined with a mucoadhesive capability in the presence of mucin (89.2 ± 1.2%) [49]. BCZ was also encapsulated in PLA nanoparticles (*d* = 205 nm) showing a spherical shape. These nanoparticles have been radiolabeled (^99m^Tc) with conjugation efficiency higher than 90% aiming early detection and tumor imaging [50].

### 2.3. Polysaccharide-Based Nanoparticles

Obtained using economical producing methods, polysaccharides are non-toxic, stable, biodegradable, biocompatible, and have structural flexibility [94,95]. The main sources of polysaccharides used to produce nanoparticles are animals (chitosan, chondroitin), microbial organisms (dextran, pullulan), plants (pectin, cellulose), and algae (alginate) [94,96].

Chitosan, composed of D-glucosamine units, is a cationic polysaccharide presenting mucoadhesive property [94,97]. These characteristics are important, since that the human biologic membranes, including cornea and conjunctiva, have a negative charge. In this sense, the positively charged groups from chitosan can electrostatically interact with these negative membranes [97]. Nanocarriers positively charged have shown higher cellular interaction and uptake, and lower phagocytosis [83,98,99].

BCZ-loaded chitosan nanoparticles (*d* = 89 and 188 nm) have been obtained with positive ζ potential (+21.6 and +6.7 mV) [51,52]. Interestingly, the nanoparticles did not aggregate after 4 h of incubation with blood plasma [51]. Furthermore, in vitro release (pH 7.4, PBS) of spherically shape nanoparticles (EE% = 38%) demonstrated a BCZ release peak after 5 days with a significant increase for 3 weeks [52].

BCZ-loaded chitosan nanoparticles (*d* = 78 nm, ζ potential = +12.6 mV, and EE% = 67.6%) have been inserted in an implant (matrix of hyaluronic acid and zinc sulfate), which mean diameter and thickness were respectively 7.49 ± 0.09 mm and 1.67 ± 0.15 mm [53]. A homogenous distribution of spherical nanoparticles has been observed in the tridimensional implant matrix. The BCZ in vitro release demonstrated a sustained release profile after 60 days from nanoparticles (83.8%) and from implant (46.7%). More recently, chitosan grafted-PEG methacrylate nanoparticles to carry BCZ have been produced [54]. The authors described mean particle size (500 nm) and ζ potential (+0.58 mV) of the blank formulation (without BCZ). Then, BCZ was included in the formulation with an EE% of 39%. In vitro controlled release has been observed (51% after 168 h) in PBS (pH = 7.4) [54].

Ternary protein-polyanion-polycation nanoparticles have been developed using BCZ, dextran sulfate nanoparticles and chitosan oligosaccharides [55]. Dextran sulfate, a mucus-penetrating polyanion with high water solubility, has hydroxyl groups modulating the incorporation of drugs into its skeleton [97]. Nanoparticles (*d* = 346 nm; ζ potential = +40.0 mV, and EE% = 73%) showed mucoadhesion in the presence of mucin [55]. The secondary and tertiary structures of BCZ were maintained after incorporation into nanoparticles.

### 2.4. Albumin Nanoparticles

Albumin nanoparticles are biocompatible natural carriers of easy preparation and reproducibility. Furthermore, these nanoparticles have a high capacity to load the drugs due to diverse functional groups in the structure [100,101]. The main albumins used are human serum albumin, bovine serum albumin, and ovalbumin [100]. Glutaraldehyde is commonly used as a crosslinking agent in the desolvation method to obtain albumin nanoparticles [100]. As an alternative to glutaraldehyde due to its reactivity with the encapsulated drug and in vivo toxicity [102,103], monoalkyl ester of the copolymer of vinyl methyl ether and maleic anhydride has been proposed. For instance, BCZ-loaded human serum albumin nanoparticles stabilized by butyl ester of poly(vinyl methyl ether/maleic anhydride) (Gantrez^®^ ES-425, 90–150 kDa) by weak bonds on the nanoparticles surface [56] have been developed. The spherical nanoparticles (*d* = 282 nm; ζ potential = −39.0 mV, and EE% = 99.5%) showed BCZ in vitro release with an initial burst (about 8%) followed by a sustained release (about 10%) within 1 h, totalizing 30% after 24 h in PBS medium.

In another study, BCZ-loaded human serum albumin nanoparticles have been produced without using a crosslinking agent [57]. The nanoparticles (*d* = 310 nm; ζ potential = −14 ± 1 mV; and EE% = 89 ± 0%) demonstrated stability due to protein-protein interactions between albumin and BCZ, which structural integrity was maintained after encapsulation. Furthermore, the stability (particle size, PDI, and ζ potential) has been also observed when incubated in PBS and cell culture medium for 24 h. BCZ in vitro release (PBS, pH 7.4) showed an initial release (about 35%) within 5 min followed by a slow and sustained release after 24 h (about 45%) [57].

The addition of PEG on the surface of nanocarriers can avoid non-specific binding with blood components [78]. PEGylated BCZ-loaded serum albumin nanoparticles (*d* = 301 ± 2 nm and ζ potential = −17 ± 1 mV) showed different characteristics compared to non-PEGylated nanoparticles (*d* = 207 ± 2 nm and ζ potential = −26 ± 1 mV) [58]. Besides, the hydrophobicity of PEGylated nanoparticles was 1.5 times lower than that of non-PEGylated nanoparticles. The PEGylated nanoparticles presented a spherical-shape with a smooth surface [59]. BCZ structural integrity was maintained after the encapsulation process with an EE% of 92%. The BCZ in vitro release (PBS, pH 7.4) showed an initial phase (about 20%) within 5 min, followed by a slow-release after 24 h (about 60%) [59].

### 2.5. Other Nanoparticles

Electrospun nanofibers have been developed as drug delivery systems providing high surface area for encapsulation of hydrophilic or hydrophobic drugs [104]. Electrospinning uses a high voltage to produce nanosized polymer fibers from a polymer solution or melt liquid [104,105]. BCZ-loaded PCL core-shell nanofibers have been produced using different pH conditions (6.2 or 8.3) showing respectively mean diameters of 520 and 469 nm, and EE% of 72.6% and 63.1% [60]. Biphasic release profile with BCZ release of 60.6 ± 7.3% after 19 days has been observed for the nanofibers prepared at pH 6.2, while those produced at pH 8.3 showed a more prolonged and monophasic BCZ release reaching 55.6 ± 16.8% after 60 days. In the latter, BCZ remained intact during the production process and release over 2 months. In contrast, BCZ released from the former nanofibers (pH 6.2) degraded losing its bioactivity [60].

PCL and gelatin core-shell nanofibers loading BCZ at different concentrations (0.5, 1, 2, and 4 wt%) showed similar diameters of nanofibers (224, 244, 272, and 255 nm, respectively). BCZ was cumulative released from the nanofibers in a deionized water medium, proportional to the amount of BCZ initially incorporated into the nanofibers within 6 days [61]. Self-associated BCZ nanoparticles have been prepared at the isoelectric point of BCZ (pH 8.4) using nonionic surfactants (polysorbate 80, polysorbate 20, or polyoxyethylene-10-oleyl-ether) to stabilize and protect the BCZ from denaturation and degradation [62]. The increase of nonionic surfactant concentration caused an increase of negative potential due to the preferential adsorption on the antibody positive charges. BCZ loaded in the nanoparticles maintained the β-sheet secondary structure retaining its bioactivity [62].

A thermo-responsive nanogel has been developed using methoxy-PEG-block-PLGA forming a spherical core-shell micellar structure (*d* = 26 to 40 nm) [63]. The micelles showed a fast and reversible sol-gel phase transition behavior (increasing the temperature from 0 to 60 °C). BCZ incorporated in nanogel (25 wt%) demonstrated a sustained release (65% within 30 days). BCZ-loaded ^99m^Tc-radiolabeled D-α-tocopheryl PEG succinate (TPGS)-based nanomicelle (*d* = 11 ± 1 nm) has been developed as a theranostic agent [64]. In another study, BCZ was co-loaded with erlotinib in hyaluronic acid-modified lipid-polymer hybrid nanoparticles (*d* = 121.7 nm; ζ potential = −21.2 mV, and EE% = 82.1%) [65]. A pH-sensitive adipic acid dihydrazide was added to this formulation. Spherical nanoparticles with smooth surface showed stable particle size, ζ potential, EE%, and drug loading after 3 months (storage at 4 °C). A faster BCZ release was observed at pH 5.5 than at pH 7.4 due to the pH-sensitive characteristic of the formulation.

### 2.6. Functionalized Nanocarriers with BCZ

Antibodies can be conjugated on the surface of nanocarriers to improve the specificity of delivery to target tissues or specific cell antigens [32,106]. In a recent study, we decorated the surface of chitosan-coated lipid-core nanocapsules with BCZ by forming an organometallic complex with gold-III (*d* = 183 nm; ζ potential = +18.5 mV) [66]. Another study reported the development of BCZ-coated PLA nanoparticles (*d* = 265 nm) inserted into porous PLGA microparticles (11.61 µm) [67]. In this case, BCZ was released showing a burst phase (21%) and a cumulative release of about 81% after 120 days in PBS (pH 7.4).

A synergistic effect can be obtained by the association of encapsulated drugs and antibodies functionalized onto the surface of nanoparticles [32]. In this sense, dexamethasone-loaded BCZ-PLGA/PEI nanoparticles have been obtained either by electrostatic interactions or by covalent binding [68]. BCZ structural stability was improved when the conjugation was based on electrostatic interactions to form the nanoparticles (*d* = 217.7 nm; ζ potential = +0.85 mV; conjugation efficiency = 56.97%). A BCZ burst release (25.2%) was observed after 12 h followed by a sustained release (60.2%) after 120 h in PBS medium [68]. In a subsequent study, cyclic Arg-Gly-Asp was added to the dexamethasone-loaded BCZ-PLGA/PEI nanoparticles (*d* = 214 nm; ζ potential = +0.30 ± 1.61 mV; and conjugation efficiency for BCZ = 83.15%). BCZ showed an initial burst release (27.3%) followed by a controlled release (56.2%) [69].

BCZ was conjugated on the surface of chemokine receptor 2 (CCR2) antagonist-loaded PEG-nanoparticles by reacting amine group in the polymer to carboxylic group of BCZ [70]. CCR2 inhibitors have been studied for the treatment of cancer and inflammatory diseases [107,108]. Nanoparticles (core size of 198 nm and shell size of 210 nm) showed a BCZ conjugation efficiency of 82% and ζ potential close to +5 mV [70]. BCZ has been conjugated on the surface of erlotinib-loaded fibrin nanoparticles by reacting amino group of fibrin with carboxyl group of BCZ (*d* = 79 nm; ζ potential = +17 mV; spherical shape with smooth surface) [71].

Triamcinolone acetonide (a corticosteroid drug) has been encapsulated in BCZ surface-functionalized lipid-nanocapsules (*d* = 102 nm; ζ potential = −19 mV) aiming the treatment of ocular diseases [72]. Thiolate BCZ was covalently bound to nanocapsules with a high percentage of surface conjugation (94 ± 5%) by using phase inversion-insertion one-step method without losing the ability to recognize VEGF [72]. In another strategy, biotinylated BCZ was bound to the surface of PEGylated liposome (*d* = 212 nm; ζ potential = +31 mV). Liposome film was hydrated with neutravidin in order to couple with BCZ [73].

Nab-Paclitaxel (Abraxane^®^) has been coated with BCZ by electrostatic interactions [74]. The nanoparticles size was directly proportional to the BCZ concentration ranging from 0.16 to 2.17 µm. The BCZ-functionalized nab-Paclitaxel showing the lower mean diameter (*d* = 160 nm) kept the ability to recognize VEGF [74]. In a subsequent study, the amino acid sequence (Val-445-Arg-472) on albumin primary structure was identified as having a nanomolar affinity with the Fab region of BCZ [75].

## 3. Pharmacological Applications

In this section we present the main applications of the organic nanocarriers containing BCZ which are the treatment of ocular diseases and the treatment and/or diagnosis of cancer (Figure 2). Other isolated applications are the treatment of atheroma, the mucosal delivery, and the toxicity studies, which are also discussed.

### 3.1. Treatment of Ocular Diseases

In normal ocular structures, there is a balance between proangiogenic and antiangiogenic factors [4]. The overexpression of VEGF has been reported as one of the contributors to several ocular diseases [109]. Aiming the intraocular drug delivery, BCZ carried in nanogel [63], liposomes [38] or albumin nanoparticles [57] demonstrated non-cytotoxicity against human-derived retinal pigment epithelial (ARPE-19) cells. The absence of a cytotoxic effect was also observed for liposomal BCZ against human umbilical vein endothelial cells (HUVEC) [38].

The bioactivity of BCZ-nanogel evaluated against chorioretinal endothelial cells (RF6A) was retained [63]. Similarly, the antiangiogenic activity of BCZ conjugated to triamcinolone acetonide-loaded lipid nanocapsules was kept when the formulation was assayed in HUVEC added of VEGF [72]. Using the chorioallantoic membrane chicken embryo (CAM) model, BCZ formulated in nanofibers [61] and dexamethasone-loaded PLGA nanoparticles [68] inhibited the growth of vessels. BCZ-loaded chitosan-coated PLGA nanoparticles [49] and BCZ encapsulated in annexin A5-associated liposomes [36] were respectively administered on chorioallantoic membrane and to rat’s eyes showing to be well-tolerated for ophthalmic use.

BCZ-loaded albumin nanoparticles administered as drops to rats remained in the eye for at least 4 h [57] and BCZ encapsulated in annexin A5-associated liposomes after topical instillation to rabbits, showed a higher BCZ concentration in the vitreous and retina/choroid than that determined for the BCZ solution [36]. In addition to topical administration, intravitreal administration was also applied in several reports. Liposomal BCZ intravitreally administered to rabbits enhanced the drug concentration-time curve and the vitreous concentration of BCZ after 3 and 42 days, in comparison to non-liposomal BCZ [35].

PLA nanoparticles in porosifying PLGA microparticles were also intravitreally administrated to rats demonstrating a sustained delivery in the vitreous region of BCZ for 45 days with detection in the vitreous humor, retina, choroid-retinal pigment epithelium, sclera, and lens after 2 months post-dosing [67]. Similarly, BCZ-loaded PLGA nanoparticles (by intravitreal via) were distributed in the retina, choroid, and sclera of rabbits, with detection up to 56 days after the administration [44]. Besides, BCZ-loaded PLGA nanoparticles increased the time to reach the maximum concentration, mean residence time, and half-life in both vitreous and aqueous humor in comparison to BCZ in solution after intravitreal administration to rabbits [44] and mice [45]. The subtenon injection of BCZ-loaded chitosan nanoparticles to the rabbit’s eyes promoted a higher amount of BCZ from nanoparticles in ocular tissues at 1, 3, 5, 7 days after injection than BCZ in solution [52].

Corneal and choroidal neovascularization is related to a variety of ocular diseases [109]. The subconjunctival injection of BCZ-loaded PLGA nanoparticles to mice almost completely inhibited corneal neovascularization after 14 days [45]. Non-PEGylated BCZ-loaded nanoparticles showed a higher antiangiogenic effect (2.4-fold) in rats than free BCZ, and lower values of cornea thickness (288 ± 85.6 µm) than PEGylated nanoparticles (431 ± 38.6 µm) [58]. In rabbits, BCZ-bearing dexamethasone-loaded PLGA nanoparticles showed a higher inhibitory effect in the neovascularization than free drugs [68]. The addition of cyclic Arg-Gly-Asp in those nanoparticles was also effective against choroidal neovascularization (reduction in choroidal neovascularization leakage area and VEGF expression) [69].

Diabetic retinopathy is a serious complication of diabetes characterized by retinal neovascularization [109]. BCZ carried in chitosan nanoparticles (intravitreal injection) [51] or chitosan grafted-PEG methacrylate nanoparticles (intraocular injection) [54] was effective as an antiangiogenic treatment in animal models (rats and rabbits, respectively) of diabetes. The retinal neovascularization in mice was more inhibited after intravitreal injection of BCZ-loaded PLGA than the free BCZ administration [45].

### 3.2. Cancer Therapy and Diagnosis

#### 3.2.1. In Vitro Studies

The tumoral microvasculature network provides the oxygen and nutrients supply necessary for cancer development; in this sense, the block of VEGF by BCZ can lead to a decrease in tumoral angiogenesis [110]. BCZ in nanoparticles [62], in solid lipid nanoparticles [41], and in PLGA nanoparticles [46] retained in vitro anti-VEGF activity evaluated against HUVEC. This bioactivity of BCZ-loaded nanoparticles was maintained for 6 months at different storage conditions (4, 25, and 40 °C) [47].

BCZ nanoparticles were cytotoxic against human non-small cell lung carcinoma cell line (A549) with a median inhibitory concentration (IC50) of 1.8 µM and higher internalization (3-fold) than in a normal lung fibroblast cell (MRC-5) [62]. BCZ-loaded PLA nanoparticles decreased survival of A549 cells and human breast cancer cells (MCF-7 and MDA-MB-231) cells [50]. Also using A549 cells, BCZ conjugated in erlotinib-loaded fibrin nanoparticles demonstrated higher cytotoxicity (IC50 = 0.84 µM) and apoptosis (61.7 ± 1.3%) than free erlotinib (IC50 = 2.3 µM; 18.35 ± 2.1%); causing a gradual decrease in G0/G1 phase, an increase in sub-G0/G1 phase, and a marked intracellular uptake [71]. Similarly, BCZ and erlotinib co-loaded in lipid-polymer hybrid nanoparticles were more cytotoxic in comparison to the free drugs against two human non-small cell lung cancer cell lines (A549 and H1975) with marked cellular uptake (about 70%) in A549 cells [65].

In melanoma cell lines (A2058 and B16-F10), the nanoemulsion containing the association of BCZ, temozolomide, and rapamycin was fast internalized with higher cytotoxicity against melanoma cell lines (PCF-2, JR-8, A2058, and B16-F10) than the free drugs [42]. In human pancreatic cancer cells (Capan-1, HPAG-II, and PANC-1) and endothelial cells (MS1-VEGF and HMEC-1), BCZ-conjugated PEGylated liposomes were cytotoxic with an improvement in cellular uptake of liposomes [73]. Similarly, liposomes with BCZ and a chromophore (benzoporphyrin derivative monoacid A) enhanced the uptake of BCZ from nanoparticles and cytotoxicity against human pancreatic ductal adenocarcinoma (PDAC) [40].

Using a model of the human blood-brain barrier (hCMEC/D3 cell line), BCZ from solid lipid nanoparticles was permeated through of hCMEC/D3 cells monolayer, while BCZ in solution was almost unable to cross the barrier [41]. In a recent report, we demonstrated that BCZ-functionalized-chitosan-coated lipid-core nanocapsules (BCZ-MLNC) tested against rat glioma (C6) showed decreased cell viability with a IC50 of 30 nmol L^−1^. Furthermore, BCZ-MLNC showed concentration and time-dependent cellular internalization [66].

#### 3.2.2. In Vivo Studies

In CAM model, BCZ-MLNC caused a higher antiangiogenic effect than BCZ in solution. Dosage decreases of 5.6 times and 2.9 times, respectively in the absence and presence of VEGF, were observed compared to BCZ solution [66]. Using a glioblastoma model in mice, BCZ-loaded PLGA nanoparticles reduced more the tumor growth than the control group with a decrease in the VEGF mRNA expression. BCZ was quantified only in the brain for the BCZ-loaded nanoparticles group; meanwhile BCZ was quantified in the lung and liver after free BCZ administration. Lung toxicity was noted only after free BCZ treatment [48].

In a non-small cell lung cancer model in mice, BCZ and erlotinib co-loaded lipid-polymer hybrid nanoparticles reduced the tumor volume compared with the free drugs (about 3-fold) and control (about 5-fold) groups [65]. In a mouse melanoma model, the nanoemulsion with BCZ, temozolomide, and rapamycin exerted significant effects on tumor volume and microvessel density, number of positive cells for Ki-67, secretion of interleukin 10, and interferon-gamma in comparison to the control group [42]. In another melanoma model in mice, the target with BCZ of Abraxane^®^ reduced the tumor size, increased the median survival (33 days) and the paclitaxel tumoral concentration [74].

In a model of pancreatic cancer in mice, the BCZ nanocarried in liposomes increased the tumor-targeting [73], decreased the uptake by the spleen [73], and reduced the tumor size combined with photodynamic therapy (33% of animals with complete reduction) [40]. The combination of liposomal BCZ with doxorubicin-loaded immunoliposomes in a human breast cancer mouse model decreased the tumor size and the doxorubicin toxicity comparing to the treatment with only immunoliposomes [37].

In a model of colorectal cancer in mice, PEGylated BCZ-loaded albumin nanoparticles reduced the tumor growth, metabolic tumor volume, and total glycolysis in comparison with BCZ in solution and control groups. The tumoral angiogenesis expression was lower in mice treated with nanoparticles than in the control group. Meanwhile, the intratumoral levels of BCZ from nanoparticles were higher than BCZ in solution (about 4-fold) [59]. Aiming the diagnosis of gastrointestinal stromal tumors, BCZ-loaded PLA nanoparticles labeled with ^99m^Tc administrated to rats were highly uptake by the liver and kidney with a moderate uptake by the tumor and the spleen [50]. Similarly, radiolabeled BCZ-TPGS based nanomicelles administrated to mice were found in the lung, liver, and kidneys with a high tumor/blood ratio [64].

### 3.3. Other Applications

Aiming the atheroma treatment, BCZ-loaded echogenic liposomes were developed as a targeted ultrasound contrast agent. Those liposomes inhibited VEGF expression and cell proliferation on HUVEC with a more evident inhibition after the ultrasound application [39]. BCZ-loaded dextran sulfate nanoparticles inhibited the angiogenesis in the CAM assay in a way more pronounced and constant reduction over time in blood vessels compared to free BCZ. This formulation was produced for the mucosal delivery [55].

The acute and chronic toxicity of BCZ-functionalized CCR2 antagonist-loaded PEG nanoparticles were evaluated in rats to determine a safe dose [70]. BCZ and CCR2 antagonist increased the risk of injury in the glomerular filtration barrier and hepatocytes in a dose-dependent manner. At high doses of BCZ (5 mg kg^−1^) and CCR2 antagonist (0.001 mg kg^−1^), the authors noted histological changes, high levels of serum creatinine and urea nitrogen. The low doses of BCZ (1.25 mg kg^−1^) and CCR2 (0.00025 mg kg^−1^) did not cause these effects [70].

## 4. Limitations and Advantages of BCZ Nanocarriers

**Figure 3:**
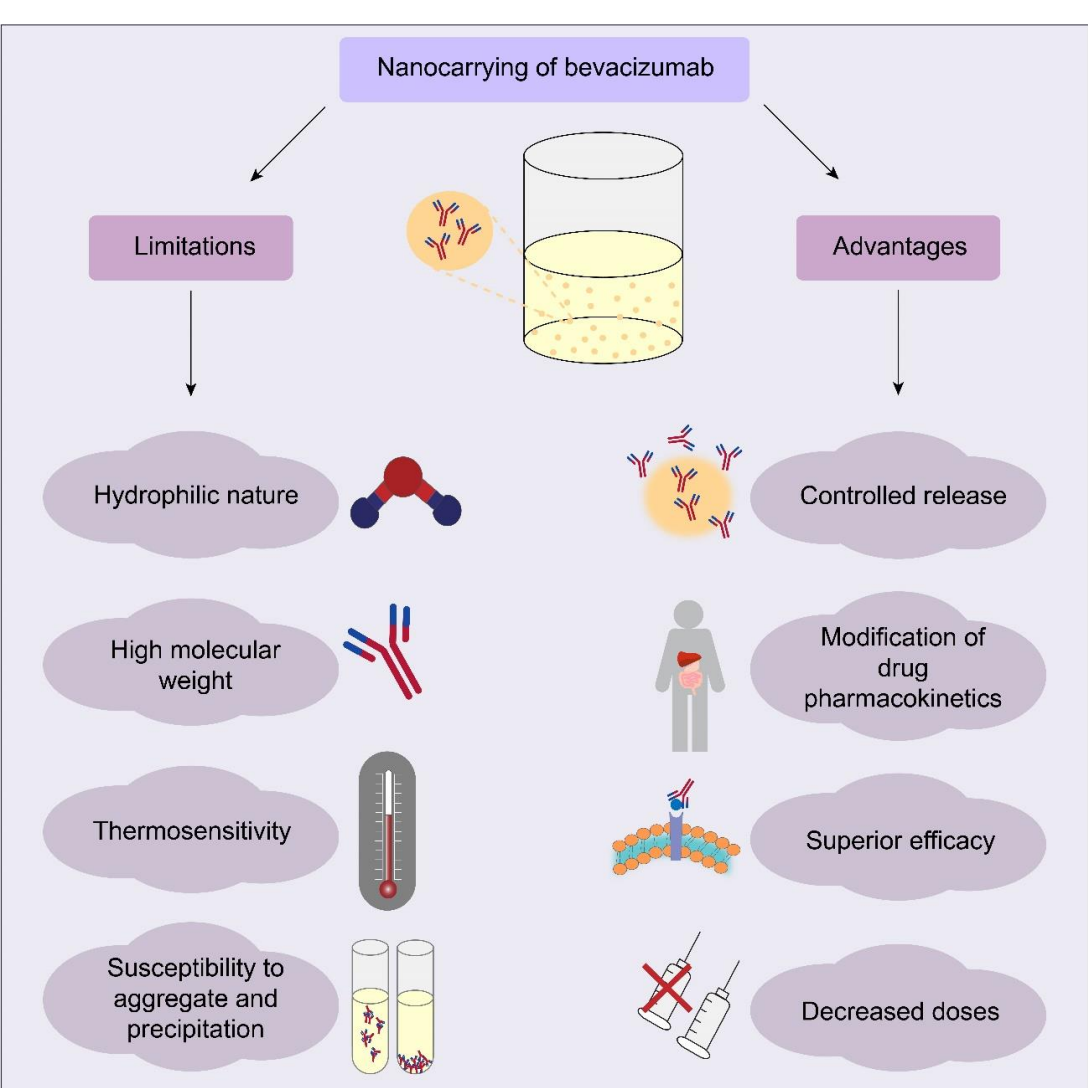
Main limitations and advantages in nanocarrying of BCZ.

BCZ nanocarriers can present limitations and advantages (Figure 3). The BCZ nanoencapsulation using organic materials can be limited due to its high molecular weight (149 kDa) and hydrophilic nature. The loading capacity depends on the drug solubility or affinity for the components of the formulation. To circumvent this limitation, the strategy of synthesizing a BCZ conjugate by the hydrophobic ion-pairing technique was proposed in some studies [41,42]. In the production of polymeric nanoparticles, the use of solvent evaporation or solvent emulsification-evaporation methods results in low EE% of hydrophilic drugs due to their diffusion to the aqueous phase [111,112]. Thus, the use of multiple emulsions (oil in water) in water (water/oil/water) was a strategy to improve the EE% of BCZ, resulting in values higher than 80% [43,45,46]. An EE% of almost 100% (99.5 ± 1.0%) was obtained for BCZ when encapsulated in human serum albumin nanoparticles and stabilized by a copolymer [56]. Comparing a similar formulation using glutaraldehyde as crosslinking agent in nanoparticles, a significant decrease in the BCZ entrapment (0.11 ± 0.3%) was observed. Possibly, glutaraldehyde reacted with BCZ by aldol condensation or Michael-type addition [56]. BCZ-loaded albumin nanoparticles produced without a crosslinking agent resulted in a high EE% (89 ± 0%), probably due to protein-protein interactions between BCZ and albumin [57]. Nanocarriers with BCZ-functionalized-surface also demonstrated high values of conjugation efficiency [68,69,70,72].

During the process of nanoencapsulation or surface conjugation, BCZ is susceptible to aggregation or precipitation and consequently, loss of its bioactivity. The choice of production method is essential to maintain the structural and functional stability of BCZ. The interfacial adsorption was reported as a problem in BCZ destabilization related to the emulsion solvent evaporation method and the albumin addition during the emulsification process, resulting in an effective stabilization of BCZ due to its surface-active properties [43]. To avoid the BCZ exposition to organic solvents or to a sonication process, BCZ-coated PLA nanoparticles encapsulated inside porous PLGA microparticles were produced by lyophilization, supercritical infusion, and pressure quench technology [67]. The maintenance of BCZ structure was observed in several reports after nanoencapsulation or surface-conjugation [47,55,62,68,74].

BCZ thermal stability is another factor that must be considered, for example, the BCZ thermal stability was determined before choosing the lipid mixture and temperature used in liposome production [40]. In another study, a fatty acid with a Krafft point below 60 °C was used due to the thermosensitivity of BCZ [41]. The maintenance of BCZ structural characteristics after encapsulation in liposomes and under thermal stress was proved [38]. Trehalose was added to PLGA nanoparticles to maintain the physical and chemical stability of BCZ. Sugars as trehalose are common protein stabilizers against dehydration, freezing, and thermal stress by a reduction of local and global mobility due to interactions of the sugar with the protein [113].

The pH control also is crucial to maintain BCZ stability during the production process and after release. BCZ-loaded PCL core-shell nanofibers [60] or BCZ nanoparticles [62] produced at the isoelectric point of BCZ remained intact the antibody structure. The antiangiogenic activity was maintained after encapsulation or surface conjugation of BCZ analyzed in vitro against cells lines [39,41,45,47,62] or in CAM model [55,61,66,68]. In animal models, BCZ encapsulated or surface conjugate demonstrated efficacy in the treatment of corneal neovascularization [45,58], choroidal neovascularization [68], antiangiogenic in a model of diabetes [54], non-small cell lung cancer [65], glioblastoma [48], melanoma [74], pancreatic adenocarcinoma combined with photodynamic therapy [40] and colorectal cancer [59].

BCZ has unfavorable pharmacokinetics, limiting the tumor penetration and permanence in ocular tissues. In this way, the controlled release of BCZ was a clear advantage observed in the majority of reports revised in this study. Therefore, these nanocarriers can decrease the multiple administrations by parenteral or intravitreal route, improving the patient compliance to treatment. Furthermore, nanoformulations with BCZ presented good tolerability [35,49,52] and hemocompatibility [38,54,65,71]. The intravitreal injection of nanoparticles with BCZ did not cause ocular toxicity in rabbits [44] neither in mice [45] or in rats [36]. Also, no toxicity was noted after intravenous [74] or intranasal [48] administration of nanoparticles with BCZ to mice.

## 5. Conclusions

Despite difficulties in working with antibodies, these nanocarriers provided superior advantages in the protection against degradation and bioactivity maintenance. The majority of reports analyzed in this review attended to minimal parameters in physicochemical characterization. However, some studies did not show data of size, polydispersity, or ζ potential, which are considered basic parameters in the analysis of nanoparticulate systems. Moreover, there is a lack of well-delineated in vivo toxicity studies, the majority of reports with in vivo experiments focused on the evaluation of therapeutic efficacy and pharmacokinetics. Although these nanocarriers have the potential to become a new therapy for ocular diseases and cancer, there is still a vast field of research unexplored on the subject to confirm their efficacy in therapeutics.

## Figures and Tables

**Table 1:** General characteristics findings from studies of organics nanocarriers with BCZ.

Drug Delivery System	Co-Encapsulated Drug	Surface Modification	Preparation Method	Application	Reference
Multilamellar liposomes	-	-	Dehydration-rehydration	Ocular diseases treatment	[35]
Unilamellar liposomes	Annexin A5	-	Lipid-film hydration	Ocular diseases treatment	[36]
Unilamellar liposomes	-	-	Solvent evaporation and film formation	Breast cancer treatment	[37]
Liposomes	-	-	Thin-film evaporation and hydration		[38]
Liposomes	-	-	Lyophilization-rehydration	Atheroma treatment	[39]
Unilamellar liposomes	Benzoporphyrin derivative	-	Freeze-thaw	Photodynamic therapy in pancreatic cancer	[40]
Solid lipid nanoparticles	-	-	Fatty-acid coacervation	Glioblastoma treatment	[41]
Nanoemulsion (Intralipid^®^)	Temozolomide and rapamycin	-	-	Melanoma treatment	[42]
PLGA nanoparticles	-	-	Double-emulsion solvent evaporation	Retinal and choroidal neovascularization treatment Ocular neovascularization treatment Corneal and retinal neovascularization treatment	[43,44,45]
PLGA nanoparticles	-	-	Double-emulsion solvent emulsification-evaporation	Glioblastoma treatment	[46,47,48]
Chitosan-coated PLGA nanoparticles	-	-	Double-emulsion solvent evaporation	Ocular diseases treatment	[49]
PLA nanoparticles	-	-	Double emulsification-solvent evaporation	Gastrointestinal stromal tumors diagnosis	[50]
Chitosan nanoparticles	-	-	Emulsification-evaporation method Ionic gelation	Diabetic retinopathy Ocular tolerability Choroidal neovascularization treatment	[51,52,53]
Chitosan grafted-PEG methacrylate nanoparticles	-	-	Double crosslinking (ionic and covalent) in reverse emulsion	Ophthalmic drug delivery system	[54]
Dextran sulfate nanoparticles	-	Chitosan oligosaccharides	Self-assembly	Mucosal delivery	[55]
Albumin nanoparticles	-	-	Desolvation	Corneal neovascularization treatment Ophthalmic drug delivery system	[56,57]
Albumin nanoparticles	-	PEG	Desolvation	Corneal neovascularization treatment Colorectal cancer treatment	[58,59]
Nanofibers	-	-	Electrospinning Coaxial electrospinning technique	Age-related macular degeneration treatment	[60,61]
Nanoparticles	-	-	Nanoprecipitation	Non-small cell lung cancer treatment	[62]
Thermo-responsive nanogel	-	-	Ring-opening polymerization and crosslinking	Intraocular drug delivery	[63]
TPGS-based nanomicelles	-	-	Dissolution	Cancer theranostic agent	[64]
Lipid-polymer hybrid nanoparticles	Erlotinib	-	Acylation reaction and sonication	Non-small cell lung cancer treatment	[65]
Chitosan-coated lipid-core nanocapsules	-	Gold-III and BCZ	Self-assembly and interfacial reactions	Glioblastoma treatment	[66]
PLA nanoparticles in porosifying PLGA microparticles	-	BCZ	Emulsion solvent evaporation	Age-related macular degeneration treatment	[67]
PLGA/PEI nanoparticles	Dexamethasone	BCZ	Emulsion solvent evaporation	Ocular diseases treatment	[68]
PLGA/PEI nanoparticles	Dexamethasone	BCZ and cRGD	Emulsion solvent volatilization	Age-related macular degeneration treatment	[69]
PEG nanoparticles	CCR2 antagonist	BCZ	-	Toxicity evaluation	[70]
Fibrin nanoparticles	Erlotinib	BCZ	Wet precipitation	Non-small cell lung cancer treatment	[71]
Lipid nanocapsules	Triamcinolone acetonide	BCZ	Phase inversion temperature	Ocular diseases treatment	[72]
Liposomes-PEG	-	BCZ	Thin-film evaporation and hydration	Pancreatic cancer treatment	[73]
Albumin nanoparticles (nab-paclitaxel, Abraxane^®^)	Paclitaxel	BCZ	-	Melanoma treatment	[74,75]

Abbreviations: BCZ, bevacizumab; CCR2, chemokine receptor 2; cRGD, Arg-Gly-Asp cyclic; PEG, poly(ethylene glycol); PEI, polyethylenimine; PLA, polylactic acid; PLGA, poly(lactic-co-glycolic acid); TPGS, d-α-tocopheryl poly(ethylene glycol) succinate.

**Table 2:** Findings of size, PDI, ζ potential, and entrapment efficiency from studies of organics nanocarriers with BCZ.

Drug Delivery System	Size (nm)	PDI	ζ Potential (mV)	Entrapment or Conjugation Efficiency (%)	Initial BCZ Amount	Reference
Liposomes	-	-	-	45.5 ± 5.6	25 mg mL^−1^	[35]
Liposomes	163 ± 73	0.20	−7.2 ± 0.6	25	50 mg mL^−1^	[36]
Liposomes	152.3 ± 9.3	-	−22.6 ± 3.1	37	200 mM	[37]
Liposomes	141.5 ± 45.8	0.36	−0.4	47.6 ± 7.0	-	[38]
Liposomes	893 ± 105	-	-	32.1 ± 6.6	0.8 mg mL^−1^	[39]
Liposomes	120 ± 8.7	-	+15.0 ± 0.3	60 to 80	6–9 µM	[40]
Solid lipid nanoparticles	515.6 ± 113.6	-	-	<30	2.5 mg	[41]
Nanoemulsion	262.6 ± 15	-	−33.1 ± 4.5	47 ± 5	1 mg mL^−1^	[42]
PLGA nanoparticles	197 ± 18	0.16 ± 0.04	−26.4 ± 2.9	82.4 ± 3.6	1 mg	[43]
PLGA nanoparticles	190 ± 29	0.17 ± 0.05	−24.5 ± 3.1	84.1 ± 4.2	1 mg	[44]
PLGA nanoparticles	~133	-	-	~80	5 mg	[45]
PLGA nanoparticles	198.6 ± 5.4	0.16 ± 0.03	−20.8 ± 1.4	82.5 ± 0.6	2 mg	[46]
PLGA nanoparticles	208 to 238	0.09 to 0.14	−6.4 to 3.4	88 ± 5	2 mg	[47]
PLGA nanoparticles	185 ± 3	0.06 ± 0.02	−2.5 ± 0.3	82.5 ± 0.6	2 mg	[48]
Chitosan-coated PLGA nanoparticles	222.3 ± 7.5	0.19 ± 0.08	+32.8	69.3 ± 1.3	1 mg	[49]
PLA nanoparticles	205 ± 1	0.06 ± 0.01	-	-	-	[50]
Chitosan nanoparticles	88.9 ± 106.7	-	+21.6 ± 2.4	-	-	[51]
Chitosan nanoparticles	188 ± 10	0.25	+6.7	38.2	12.5 mg	[52]
Chitosan nanoparticles	78.5 ± 1.9	0.13 ± 0.05	+12.6 ± 1.51	67.6 ± 6.7	1 mg	[53]
Chitosan-PEG-methacrylate nanoparticles	500	-	+0.6	39	25 mg	[54]
Dextran sulfate nanoparticles	346 ± 2	0.33	+40.0 ± 0.5	73 ± 2	0.5 mg mL^−1^	[55]
Albumin nanoparticles	282 ± 4	0.12	−39.0 ± 0.9	99.5 ± 1.0	15 mg	[56]
Albumin nanoparticles	310 ± 3	0.14 ± 0.02	−14 ± 1	89 ± 0	-	[57]
Albumin nanoparticles	207 ± 2	0.10 ± 0.01	−26 ± 1	-	15 mg	[58]
Albumin nanoparticles	301 ± 2	0.13 ± 0.03	−17 ± 1	92 ± 4	15 mg	[59]
Nanofibers	pH 6.2: 520 ± 120 pH 8.3: 469 ± 83	-	-	pH 6.2: 72.6 ± 1.1 pH 8.3: 63.2 ± 0.3	12.5 mg	[60]
Nanofibers	224 ± 44 to 272 ± 86	-	-	-	1.25 to 10 mg	[61]
Nanoparticles	P80: 395.4 ± 2.2 P20: 334.4 ± 2.6 P10OE: 365.6 ± 4.1	<0.20	P80: −13.7 ± 0.7 P20: −10.5 ± 0.5 P10OE: −9.7 ± 0.4	-	5 mg mL^−1^	[62]
Thermo-responsive nanogel	26.1 to 39.7	-	-	-	12.5 mg mL^−1^	[63]
TPGS-based nanomicelles	11 ± 1	0.20 ± 0.01	-	-	2.5 mg mL^−1^	[64]
Lipid-polymer hybrid nanoparticles	121.7 ± 3.9	0.15 ± 0.02	−21.2 ± 2.9	82.1 ± 2.7	10 mg	[65]
Chitosan-coated lipid-core nanocapsules	183 ± 21	0.22 ± 0.04	+18.5 ± 1.9	-	200 µg mL^−1^	[66]
PLA nanoparticles in porosifying PLGA microparticles	265 ± 9	-	-	-	2.5 mg	[67]
PLGA/PEI nanoparticles	217.7 ± 5.3	0.28 ± 0.05	+0.9 ± 0.4	85.6 ± 0.3	-	[68]
PLGA/PEI nanoparticles	213.8 ± 1.5	0.15 ± 0.04	+0.3 ± 1.6	83.2 ± 1.7	-	[69]
PEG nanoparticles	210	-	~ + 5	82	25 mg mL^−1^	[70]
Nanofibrin	79	-	+17	-	6.25 mg	[71]
Lipid nanocapsules	102 ± 15	0.15	−19 ± 6	94 ± 5	2.5 mg	[72]
Liposomes-PEG	212 ± 35	-	+31 ± 4	-	1 mg	[73]
Albumin nanoparticles (nab-paclitaxel, Abraxane^®^)	~160	-	-	-	4 mg mL^−1^	[74]
Albumin nanoparticles (nab-paclitaxel, Abraxane^®^)	158.9	-	-	-	4 mg mL^−1^	[75]

Abbreviations: PEG, poly(ethylene glycol); PEI, polyethylenimine; PLA, polylactic acid; PLGA, poly(lactic-co-glycolic acid); P10OE, polyoxyethylene−10-oleyl-ether; P20, polysorbate 20; P80, polysorbate 80; PDI, polydispersity index; TPGS, d-α-tocopheryl poly(ethylene glycol) succinate.
